# Introduction to the Epidemiologic Considerations, Analytic Methods, and Foundational Results From the Pneumonia Etiology Research for Child Health Study

**DOI:** 10.1093/cid/cix142

**Published:** 2017-05-27

**Authors:** Katherine L. O’Brien, Henry C. Baggett, W. Abdullah Brooks, Daniel R. Feikin, Laura L. Hammitt, Stephen R. C. Howie, Maria Deloria Knoll, Karen L. Kotloff, Orin S. Levine, Shabir A. Madhi, David R. Murdoch, J. Anthony G. Scott, Donald M. Thea, Scott L. Zeger

**Affiliations:** 1 Department of International Health, Johns Hopkins Bloomberg School of Public Health, Baltimore, Maryland;; 2 Global Disease Detection Center, Thailand Ministry of Public Health–US Centers for Disease Control and Prevention Collaboration, Nonthaburi;; 3 Division of Global Health Protection, Center for Global Health, Centers for Disease Control and Prevention, Atlanta, Georgia;; 4 International Centre for Diarrhoeal Disease Research, Bangladesh (icddr,b), Dhaka and Matlab;; 5 Division of Viral Diseases, National Center for Immunizations and Respiratory Diseases, Centers for Disease Control and Prevention, Atlanta, Georgia;; 6 Kenya Medical Research Institute-Wellcome Trust Research Programme, Kilifi;; 7 Medical Research Council Unit, Basse, The Gambia;; 8 Department of Paediatrics, University of Auckland, and;; 9 Centre for International Health, University of Otago, Dunedin, New Zealand;; 10 Division of Infectious Disease and Tropical Pediatrics, Department of Pediatrics, Center for Vaccine Development, Institute of Global Health, University of Maryland School of Medicine, Baltimore;; 11 Bill & Melinda Gates Foundation, Seattle, Washington;; 12 Medical Research Council: Respiratory and Meningeal Pathogens Research Unit, and; 13 Department of Science and Technology/National Research Foundation: Vaccine Preventable Diseases Unit, University of the Witwatersrand, Johannesburg, South Africa; 14 Department of Pathology, University of Otago, Christchurch, and; 15 Microbiology Unit, Canterbury Health Laboratories, Christchurch, New Zealand;; 16 Department of Infectious Disease Epidemiology, London School of Hygiene & Tropical Medicine, United Kingdom;; 17 Center for Global Health and Development, Boston University School of Public Health, Massachusetts;; 18 Department of Biostatistics, Johns Hopkins Bloomberg School of Public Health, Baltimore, Maryland

**Keywords:** pneumonia, etiology, children, case-control analysis, PERCH.

## PNEUMONIA MORTALITY REDUCTIONS OF PAST 3 DECADES

**Figure 1.**
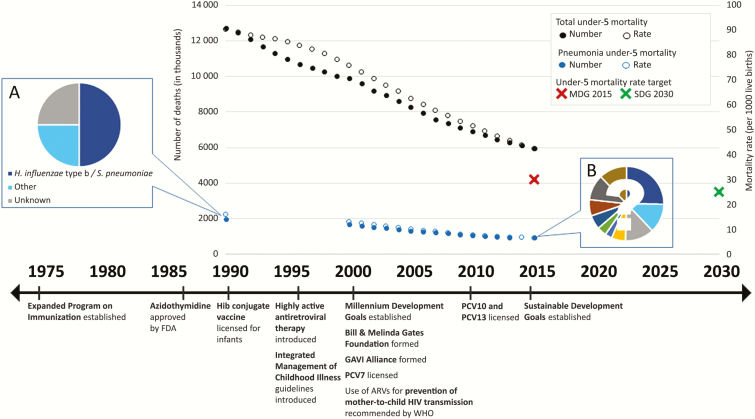
Annual under-5 all-cause and pneumonia mortality rates with global targets and pivotal health interventions, by time. *A*, The conceptual pneumonia mortality etiology distribution from the preconjugate vaccine era and *B*, the unknown etiologic distribution of the present and future. Abbreviations: ARVs, antiretrovirals; FDA, US Food and Drug Administration; Hib, *Haemophilus influenzae* type b; MDG, Millenium Development Goal; PCV, pneumococcal conjugate vaccine; SDG, Sustainable Development Goal; *S. pneumoniae, Streptococcus pneumoniae*; WHO, World Health Organization.

Over the last 20–30 years, enormous reductions have occurred in the absolute and relative burden of pneumonia mortality in young children around the world. Only 20 years ago, when the population of young children was approximately 625 million, approximately 1.7 million young children died from pneumonia before their 5th birthday (Figure 1) [1–4]. Mortality from pneumonia among children aged <5 years fell to 921 000 in 2015, whereas the population of young children rose to >670 million [1, 2, 5]. This remarkable improvement in child survival and health has resulted from advances in social conditions and economic development [6] but has also been influenced by at least 4 pivotal innovations: (1) the development of a global vaccination program, the World Health Organization’s Expanded Program on Immunizations (begun in 1974), which created the architecture around which country investments, donor funding, program strategies, and outcome measurements could be envisioned and implemented; (2) the global consensus to focus funding, programs, and momentum on 6 development targets articulated by the United Nations General Assembly through the Millennium Development Goals (MDGs, agreed upon in 2000) with MDG4 targeting child survival; (3) the advent of large, health-focused nongovernmental organizations; and (4) the founding of the Global Alliance for Vaccines and Immunization (the Gavi Alliance, formally launched at the World Economic Forum in January 2000), a multilateral funding organization that has allowed for an unprecedented pace of introduction and expanded use of life-saving vaccines in low-income countries. In part, as a result of this multidimensional, multisectoral consensus approach enacted through critical large-scale investments in prevention, protection, and treatment, pneumonia mortality has fallen substantially in many parts of the world because the most fatal of the pathogens and the underlying conditions that put children at risk are being targeted.

## NEW EVIDENCE BASE NEEDED TO REDUCE PNEUMONIA BURDEN FURTHER

Over the past 3 decades, much effort has been placed on developing, licensing, introducing, and optimizing coverage with new vaccines and on developing and implementing evidence-based case management strategies for childhood pneumonia. More recently, the focus has extended to reducing the underlying conditions that put children at risk of pneumonia mortality, including reducing human immunodeficiency virus (HIV) infection through prevention of mother-to-child transmission, preventing and treating malnutrition and undernutrition, reducing household and outdoor air pollution exposure, and ensuring that prevention and treatment services are accessible when and where they are needed. The reduction in pneumonia burden over the last decades has not been equal across all pathogens, countries, populations, or communities within countries. Selected pathogens have been targeted by vaccines (eg, *Haemophilus influenzae* type b [Hib], pneumococcus, measles, pertussis), and childhood disease burden from these pathogens has therefore been reduced disproportionately compared with other pathogens. Consequently the current etiologic distribution of pneumonia-causing pathogens is not just a smaller replica of the etiologic distribution from 20–30 years ago. With pneumonia still the leading cause of childhood deaths, the importance of quantifying and characterizing the contribution of pathogens causing those deaths is key (Figure 1).

Furthur advancing the reduction of pneumonia burden among young children means moving beyond the “low-hanging fruit” interventions. Innovation is needed not only to maintain the pace of pneumonia mortality reduction of the past decade but also to accelerate that pace, with the aim of achieving global targets for health [7]. With 48% of pneumonia deaths occurring in 5 countries in Asia and Africa (India, Nigeria, Pakistan, Democratic Republic of Congo, and China), which together account for only 41% of the world’s population aged <5 years [1, 2], an understanding of geographic variability in pneumonia is also important.

Motivated by this global vision for accelerating the pace of change, and recognizing that to achieve that vision advances would be needed in pneumonia prevention and control, a simple question was posed to the technical community by the Bill & Melinda Gates Foundation several years ago. In a world where existing tools to reduce pneumonia mortality have been deployed, including the introduction and widespread use of available vaccines, what will be the remaining causes of pneumonia and targets for prevention and treatment? The answer to this question will inform investments in tools needed to create an accelerated downward inflection in the pneumonia and child mortality rates and achieve the goals set by the world for child survival.

## ETIOLOGY OF PNEUMONIA MORTALITY IN A NEW ERA

**Figure 2.**
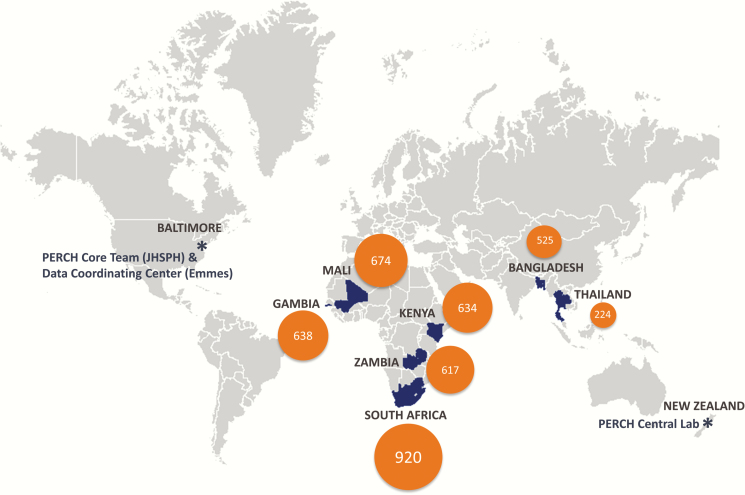
Countries where Pneumonia Etiology Research for Child Health (PERCH) study sites are located, and case enrollment numbers. Abbreviations: Emmes, The Emmes Corporation; JHSPH, Johns Hopkins Bloomberg School of Public Health.

An expert consultation on pneumonia, convened by the Bill & Melinda Gates Foundation in 2007, recommended that a large-scale study of pneumonia etiology be conducted, focusing on severe pneumonia as the closest proxy for pneumonia mortality. The Pneumonia Etiology Research for Child Health (PERCH) study was commissioned to address this recommendation; its goal is to characterize the etiology of serious pneumonia in young children in geographic settings that would provide a robust evidence base for strategies to accelerate reductions in pneumonia morbidity and mortality, particularly for children in sub–Saharan Africa and south Asia. PERCH enrolled >4000 cases and >5000 controls in 7 countries (Figure 2) and is likely the most comprehensive study of the causes of pneumonia in young children yet undertaken. The study has brought together the expertise and advice of dozens of investigators from around the world. The epidemiologic basis, strategic decisions, and study methods for the PERCH case–control study were published as a supplement in 2012 [8].

The fundamental and vexing problem in pneumonia etiology work is that we cannot usually sample the tissue where the disease occurs: the lung. This contrasts sharply with the other global public health targets, including diarrhea, measles, malaria, and HIV. For pneumonia, we are stuck collecting and testing samples from multiple body fluids that are contiguous with, but distant from, the site of infection or from body fluids that are a proxy for the site of infection. Pneumonia etiology studies require analytic approaches that can integrate data from multiple sources through sound epidemiologic and statistical methods, which produce valid insights into etiology. In addition to the challenges of specimen collection, there is also imperfect understanding of the likelihood of single-pathogen versus multi-pathogen infections in the pathophysiology of pneumonia, making the validity of assumptions necessary for the analysis of pneumonia etiology data uncertain. Beyond the inability to collect specimens from the site of infection, pneumonia studies lack a clinical case definition that is both highly sensitive and specific, meaning that pneumonia studies will either miss substantial groups of children with pneumonia or will include substantial numbers of children who do not have pneumonia.

This study differs qualitatively and quantitatively from previous pneumonia etiology studies. The efforts to standardize and integrate the clinical, laboratory, epidemiologic, and analytic approaches and data management have been a cornerstone commitment of the project. That is not to say that this study is without limitations because no amount of analysis can fully overcome the challenges inherent in pneumonia research.

## CONTEXT, METHODS, AND PREPARATORY RESULTS FROM PERCH

With the articles in this supplement, we begin reporting the findings from PERCH. These articles fall into 3 categories: context, methods, and preparatory results. We provide results from specimens and tests that had not been part of standard pneumonia etiology assessments before (eg, polymerase chain reaction [PCR] density of detected pathogens in the nasopharynx, induced sputum results, C-reactive protein) to assess their inferential value. These articles also aim to provide transparency on the critical quantitative decisions we have made along the path toward the primary etiologic analysis. Several of the articles describe efforts to navigate the fundamental problem of being unable to sample the lung directly.

### Context Articles

These articles offer insights into the pneumonia etiology field leading up to the PERCH study and therefore the context within which the methods and results should be considered. The history of pneumonia etiology article [9] walks through the advances and, on occasion, the retreats made in etiology studies over the past century. Our reflections and analysis contextualize why the PERCH study was designed as it was and the vexing limitations that we aimed to overcome through its design. Over the past 100 years of pneumonia etiology work, several analytic approaches have been used, each with their own benefits and limitations, which we organize and interrogate to provide a context for the analytic approaches needed for PERCH [10]. The specific issue of determining the appropriate control group for the primary analysis was also carefully considered, especially the biases that would be introduced with the inclusion or exclusion of control subjects with respiratory symptoms [11].

### Methods Descriptions

Next we provide methods articles, which describe the implementation of the study protocol and analytic approaches that were essential for the use of the pneumonia etiology data. PERCH was committed to conducting all analyses in a fully standardized way across all 7 countries, using the same protocols, methods, equipment, and reagents so that etiology differences across geographies cannot be explained by heterogeneity in methods. Laboratory testing procedures and assessment of the clinical data were measured in as standardized a way as possible across all sites, which included extensive on-site training and monitoring [12, 13]. The chest radiographs formed a key element of the case definition for the case–control analysis. Standardized reading processes are described, along with their degree of concordance in reading [14]. The lessons and methods used in a large, observational, multicountry, multi-investigator study can help future studies adopt and further advance best practices, so in the data management article we provide a full description of the approaches we took to assure the quality of the PERCH data, including reductions in misclassifications, errors, and missing data [15]. To address the limitations of existing analytic methods, we developed a new partial latent class, Bayesian analytic approach that allows for the integration of microbiologic results from multiple body fluids and multiple tests for a single pathogen and incorporates adjustments for sensitivity and specificity. The model has been described in the statistical computational literature [16, 17], and here we provide a description of the approach from an epidemiologic perspective [10] and an analysis of the advantages conferred by the new approach [18].

### Preparatory Results

Finally we provide articles that report results from PERCH cases and controls on issues that are relevant to pneumonia etiology studies generally but also inform decisions that were needed for the PERCH main etiology analysis. These results are not unique to the study settings involved but are generalizable and applicable beyond PERCH to other pneumonia diagnostics efforts. For example, induced sputum analysis, including for tuberculosis [19–23], and pathogen density measurement [24–26] are 2 strategies that aspired to approximate more closely testing for the pathogen infecting the lung. Because blood culture for bacterial pneumonia detection is poorly sensitive, we aimed to understand the impact of pretreatment with antibiotics and specimen volume on sensitivity, which is critical for analytic adjustments [27]. To increase the sensitivity of these bacteremic detections, we assessed the value of whole-blood molecular testing on cases and also on controls to quantify the limitations of this new diagnostic test in determining etiology [28, 29]. In addition, biomarkers like C-reactive protein may help distinguish bacterial from nonbacterial causes of pneumonia [30]. Chest radiograph findings are critical for characterizing the enrolled cases across the different sites and for identifying the cases confirmed to have pneumonia syndrome. The results of the chest radiograph reading process designed to minimize misclassification and the descriptive findings among the cases, by site and case severity, are therefore key to the interpretation of the results from the main etiology analysis [31].

## CONCLUSION

The PERCH study has faced a series of critical decisions about how to handle numerous data elements, and we have been committed to basing those decisions on evidence rather than on dogma or anecdote. By clearly showing, through these articles, the evidence and rationale for the decisions we made, we hope to minimize the potential pitfalls of arbitrariness or bias in deriving overall etiologic results in our study and for others. We also aimed through these preparatory analyses to remove the “black box” that can obscure the approach to and analysis of complex data.

By publishing these articles on the context, methods, and preparatory results from PERCH, we hope to provide the pneumonia community a seat at the analytic table. We aim to provide transparency in our lines of thinking, in our interrogation of the data to inform the decisions necessary for a comprehensive analysis of pneumonia etiology, and in our inferences about what these data teach us about a complex biologic condition.

## Notes

***Author contributions.*** K. L. O. drafted the manuscript. K. L. O., H. C. B, W. A. B., D. R. F., L. L. H., S. R. C. H., M. D. K., K. L. K., O. S. L., S. A. M., D. R. M., J. A. G. S., and D. M. T. designed and supervised the study. O. S. L. and K. L. O. served as the overall study principal investigators. H. C. B, W. A. B., S. R. C. H., K. L. K., S. A. M., J. A. G. S., and D. M. T. served as the field site principal investigators. L. L. H. and D. R. F. led clinical/epidemiology, M. D. K. led data management and analysis, and D. R. M. led laboratory operations. S. Z. provided statistical expertise and led the integrated etiology analysis. All authors reviewed and approved the manuscript. K. L. O. had final responsibility for the decision to submit for publication.

***Acknowledgments.*** We offer sincere thanks to the patients and families who participated in this study. The authors gratefully acknowledge all those at the Bill & Melinda Gates Foundation who, over the duration of the PERCH study, have supported its conduct, including Richard Adegbola, Doug Holtzmann, Angela Hwang, Ann-Marie Kimball, Keith Klugman, Trevor Mundel, Gina Rabinovich, and Gail Rodgers. The authors are indebted to the members of the PERCH Expert Group (William C. Blackwelder, Harry Campbell, John A. Crump, Adegoke Falade, Menno D. de Jong, Claudio Lanata, Kim Mulholland, Shamim Qazi, Cynthia G. Whitney) who provided invaluable advice during the conduct of the data collection and the analysis period, and the members of the Pneumonia Methods Working Group (Zulfiqar Bhutta, Robert Black, Harry Campbell, Thomas Cherian, Derrick Crook, Scott Dowell, Steve Graham, Menno D. de Jong, Keith Klugman, Claudio Lanata, Shabir Madhi, Paul Martin, James Nataro, Franco Piazza, Shamim Qazi, Heather Zar) who contributed substantially to the design of the PERCH study. The authors also express their gratitude and respect for all of the members of the PERCH Study Group, whose tireless efforts at each of the field sites and on the core team to collect the data, test the specimens, and analyze the results with scrupulous attention to detail ensure the integrity of the new knowledge from this study.

***PERCH Study Group***. Johns Hopkins Bloomberg School of Public Health, Baltimore, Maryland: Katherine L. O’Brien (PI), Orin S. Levine (former PI; current affiliation Bill & Melinda Gates Foundation, Seattle, Washington), Maria Deloria Knoll (co-PI), Daniel R. Feikin (joint affiliation with Centers for Disease Control and Prevention, Atlanta, Georgia), Andrea N. DeLuca, Amanda J. Driscoll, Nicholas Fancourt, Wei Fu, Laura L. Hammitt, Melissa M. Higdon, E. Wangeci Kagucia, Ruth A. Karron, Mengying Li, Daniel E. Park, Christine Prosperi, Zhenke Wu, Scott L. Zeger. The Emmes Corporation, Rockville, Maryland: Nora L. Watson. Nuffield Department of Clinical Medicine, University of Oxford, United Kingdom: Jane Crawley. University of Otago, Christchurch, New Zealand: David R. Murdoch. ICDDR, b, Dhaka and Matlab, Bangladesh: W. Abdullah Brooks (site PI), Hubert P. Endtz, Khalequ Zaman, Doli Goswami, Lokman Hossain, Yasmin Jahan, Hasan Ashraf. Medical Research Council, Basse, The Gambia: Stephen R. C. Howie (site PI), Bernard E. Ebruke, Martin Antonio, Jessica McLellan, Eunice Machuka, Arifin Shamsul, Syed M.A. Zaman, Grant Mackenzie. KEMRI-Wellcome Trust Research Programme, Kilifi, Kenya: J. Anthony G. Scott (site PI and PERCH co-PI), Juliet O. Awori, Susan C. Morpeth, Alice Kamau, Sidi Kazungu, Micah Silaba Ominde. Division of Infectious Disease and Tropical Pediatrics, Department of Pediatrics, Center for Vaccine Development, Institute of Global Health, University of Maryland School of Medicine, Baltimore, Maryland, and Centre pour le Développement des Vaccins (CVD-Mali), Bamako, Mali: Karen L. Kotloff (site PI), Milagritos D. Tapia, Samba O. Sow, Mamadou Sylla, Boubou Tamboura, Uma Onwuchekwa, Nana Kourouma, Aliou Toure. Respiratory and Meningeal Pathogens Research Unit, University of the Witwatersrand, Johannesburg, South Africa: Shabir A. Madhi (site PI), David P. Moore, Peter V. Adrian, Vicky L. Baillie, Locadiah Kuwanda, Azwifarwi Mudau, Michelle J. Groome, Nasreen Mahomed. Thailand Ministry of Public Health–US CDC Collaboration, Nonthaburi, Thailand: Henry C. Baggett (site PI), Somsak Thamthitiwat, Susan A. Maloney (former site PI), Charatdao Bunthi, Julia Rhodes, Pongpun Sawatwong, Pasakorn Akarasewi (site co-PI, Ministry of Public Health). Boston University School of Public Health, Boston, Massachusetts, and University Teaching Hospital, Lusaka, Zambia: Donald M. Thea (site PI), Lawrence Mwananyanda, James Chipeta, Phil Seidenberg, James Mwansa, Somwe wa Somwe, Geoffrey Kwenda. Canterbury Health Laboratory, Christchurch, New Zealand: Trevor P. Anderson, Joanne Mitchell.

***PERCH Chest Radiograph Reading Panel***

**Readers:** Dr Kamrun Nahar, Dr Fariha Bushra Matin, Dr Claire Oluwalana, Dr Bernard E. Ebruke, Dr Joyce Sande, Dr Micah Silaba Ominde, Dr Mahamadou Diallo, Dr Breanna Barger-Kamate, Dr Nasreen Mahomed, Dr David P. Moore, Dr Anchalee Kruatrachue, Dr Piyarat Suntarattiwong, Dr Musaku Mwenechanya, Dr Rasa Izadnegahdar. Arbitrators: Dr Vera Manduku, Dr John DeCampo, Dr Marg DeCampo, Dr Fergus Gleeson.

***PERCH Contributors:***

**Bangladesh:** Kamrun Nahar, Arif Uddin Sikdir, Sharifa Yeasmin, Dilruba Ahmed, Muhammad Ziaur Rahman, Muhammad Yunus, Muhammad Al Fazl Khan, Muhammad Jubayer Chisti, Abu Sadat Muhammad Sayeem, Shahriar Bin Elahi, Mustafizur Rahman. The Gambia: Michel Dione, Emmanuel Olutunde, Peter Githua, Ogochukwu Ofordile, Rasheed Salaudeen, David Parker. Kenya: Shebe Mohamed, Siti Ndaa, Micah Silaba, Neema Muturi, Angela Karani, Sammy Nyongesa, Anne Bett, Daisy Mugo, Salim Mwarumba, Robert Musyimi, Andrew Brent, James Nokes, David Mulewa, Joyce Sande, John Odhiambo, Joshua Wambua, Nuru Kibirige, Caroline Mulunda, Hellen Mjalla, Norbert Katira, Karen Dama, Loice Masha, Christine Mutunga, Mwanajuma Ngama, Stephen Mangi, Riziki Anthony, Mwarua Yubu, Elijah Wakili, Benson Katana, Shoboi Mgunya, Emmanuel Mumba, Benedict Mver, George Kuria, Felix Githinji, Norbert Kihuha, Boniface Jibendi, Tahreni Bwanaali, Agustus Kea. Mali: Nana Kourouma, Aliou Toure, Mahamadou Diallo, Breana Barger-Kamate, Mariam Samake, Seydou Sissoko, Abdoul Aziz Maiga, Mariam Samake, Toumani Sidibe, Mariam Sylla, Aziz Diakite, Bassirou Diarra. South Africa: Azwidihwi Takalani, Andrea Hugo, Susan Nzenze, Ndulela Titi, Mmabatho Selela, Malebo Motiane, Minah Nkuna, Nonhlanhla Tsholetsane, Sibonsile Moya, Debra Katisi, Tondani Netshishivhe, Lerato Mapetla, Gudani Singo, Simphiwe Gasa, Cece Mgenge, Nozipho Mthunzi, Nombulelo Monedi, Tanja Adams, Shafeeka Mangera, Jeannette Wadula, Peter Tsaagane, Jenifer L. Vaughan, Sakina Loonat, Martin Hale, Sugeshnee Pather, Mariëtte Middel, Siobhan Trenor, Palesa Morailane, Ntombi Maya, Rene Sterley, Charné Combrinck, Given Malete, Lerato Qoza, Grizelda Liebenberg, Hendrik van Jaarsveld, Zunaid Kraft, Lisa-Marie Mollentze, Lourens Combrinck, Tsholofelo Mosome. Thailand: Sununta Henchaichon, Dr Tussanee Amornintapichet, Dr Somchai Chuananont, Toni Whistler, Juraiporn Ratanodom, Patranuch Sapchookul, Ornuma Sangwichian, Sirirat Makprasert, Manoon Hirunsalee, Possawat Jorakate, Anek Kaewpan, Duangkamol Siludjai, Apiwat Lapamnouysup, Dr Wantana Paveenkittiporn, Waraporn Ubonphen, Dr Peera Areerat, MsYupapan Wannachaiwong, Ms Tewa Faipet, Ms Punnat Natnarakorn, Ms Ahchanan Sacharone, Mr.Winai Makmool, Ms Kanlaya Sornwong, Ms Promporn Sansuriwong, Ms Ratchanida Potiya, Ms Wasana Hongsawong, MsWipa Matchaikhen, Ms Thatsanawan Chaiyabil, MsPiyapai Wannarach, Ms Chamaiporn Wadeesirisak, Mr. Yuttapong Norapet, Mattana Bangkung, Mr. Barameht Piralam, Sathapana Naorat, Anchalee Jatapai, Prasong Srisaengchai, Dr Leonard Peruski, MsDawan Phaensoongnoen, MsTussaaorn Klangprapan, MsNarawadee Dumrongdee, MsAtchara Srithongkham, Mr. Piyawut Noinont, Ms Pornthip Kamlee, Ms Siyapa Mongkornsuk. Zambia: Justin Mulindwa, Musaku Mwenechanya, John Mwaba, Magdalene Mwale, Julie Duncan, Kazungu Siazele, Muntanga Mapeni, Emily Hammond. Canterbury Health Laboratory, Christchurch, New Zealand: Rose Watt, Shalika Jayawardena. The Emmes Corporation, Rockville, Maryland: Mark Wolff, Megan Sanza, Omid Neyzari.

This article is published with the permission of the Director of the Kenya Medical Research Institute.

***Supplement sponsorship.*** This article appears as part of the supplement “Pneumonia Etiology Research for Child Health (PERCH): Foundational Basis for the Primary Etiology Results,” sponsored by a grant from the Bill & Melinda Gates Foundation to the PERCH study of Johns Hopkins Bloomberg School of Public Health, Baltimore, Maryland.

***Financial support.*** PERCH was supported by grant 48968 from The Bill & Melinda Gates Foundation to the International Vaccine Access Center, Department of International Health, Johns Hopkins Bloomberg School of Public Health. J. A. G. S. was supported by a clinical fellowship from The Wellcome Trust of Great Britain (098532).

***Potential conflicts of interest.*** K. L. O. has received grant funding from GSK and Pfizer and participates on technical advisory boards for Merck, Sanofi Pasteur, PATH, Affinivax, and ClearPath. M. D. K. has received funding for consultancies from Merck, Pfizer, Novartis, and grant funding from Merck. L. L. H. has received grant funding from Pfizer and GlaxoSmithKline. K. L. K. has received grant funding from Merck Sharp & Dohme. S. A. M. has received honorarium for advisory board from the Bill & Melinda Gates Foundation (BMGF), Pfizer, Medimmune, and Novartis, institutional grants from GSK, Novartis, Pfizer, Minervax, and BMGF, and speakers bureau for Sanofi Pasteur and GSK. All other authors: No reported conflicts. All authors have submitted the ICMJE Form for Disclosure of Potential Conflicts of Interest. Conflicts that the editors consider relevant to the content of the manuscript have been disclosed.
